# Modulatory role of nitric oxide in cobalt-induced stress in two lettuce (*lactuca sativa* l.) varieties: a physiological approach

**DOI:** 10.3389/fpls.2026.1731303

**Published:** 2026-02-20

**Authors:** Halil Samet, Yakup Çıkılı

**Affiliations:** 1 Department of Crop and Animal Production, Kocaeli University, Izmit Vocational School, Kocaeli, Türkiye; 2 Department of Soil Science and Plant Nutrition, Çanakkale Onsekiz Mart University, Faculty of Agriculture, Çanakkale, Türkiye

**Keywords:** accumulation, antioxidant enzymes, CO toxicity, growth, lettuce, nitric oxide, oxidative stress

## Abstract

Cobalt (Co) toxicity poses a serious constraint on plant growth by inducing oxidative stress and disrupting cellular and physiological processes. This study investigated the interactive effects of Co and sodium nitroprusside (SNP), a nitric oxide (NO) donor, on two lettuce varieties (*Lactuca sativa* L.): curly (var. *crispa*) and Romaine (var. *longifolia*) under controlled hydroponic conditions. Plants were exposed to Co and SNP treatments, and growth parameters, oxidative stress indicators, antioxidant enzyme activities, and Co accumulation in roots and shoots were evaluated. Co exposure markedly reduced shoot and root biomass and increased membrane permeability, hydrogen peroxide (H_2_O_2_), malondialdehyde (MDA), proline accumulation, and Co concentrations in both varieties. Co+SNP application partially alleviated Co-induced stress relative to Co treatment alone, as evidenced by moderated membrane permeability (MP), reduced lipid peroxidation (MDA), and modulation of antioxidant enzyme activities (CAT and APX), particularly in Romaine lettuce. In contrast, the response of curly lettuce to SNP application was limited, likely due to its higher Co accumulation and greater oxidative burden. Overall, the results demonstrate genotype-dependent responses to SNP application under high-dose Co stress. Although SNP modulated several stress-related parameters, Co accumulation in edible shoot tissues highlights potential food safety concerns. Therefore, the findings should be interpreted within the context of controlled hydroponic systems and provide insight into genotype-dependent stress responses rather than recommendations for food production in Co-contaminated environments.

## 1 Introduction

Co is not considered an essential element for plants; it is a transition metal that primarily exists in the +2 and +3 oxidation states and may act as a catalyst in Fenton reactions (Lange et al., 2017). Physiologically, Co functions as a coenzyme in several cellular processes, including vitamin B12 (cyanocobalamin) synthesis, fatty acid oxidation, and DNA synthesis (Adolfo et al., 2016; Bennet, 1994). Co has also been reported to delay leaf senescence by inhibiting ethylene biosynthesis (Pilon-Smits et al., 2009). For optimal plant growth, soil Co concentrations should range between 3.4–8.5 µM (Bennet, 1994). However, excessive Co in the growth medium can be toxic, with a reported toxicity threshold of approximately 50 µM, similar to other divalent cations such as Cu²^+^, Ni²^+^, and Zn²^+^ (Gopal et al., 2003; Chatterjee and Chatterjee, 2005; Karuppanapandian and Kim, 2013; Agnihotri et al., 2014).

Excessive Co exerts cytotoxic effects, including inhibition of mitosis, chromosome damage, endoplasmic reticulum disruption in root tips, and irregular phloem formation (Akeel and Jahan, 2020). It also induces oxidative stress, inhibits photosynthesis, and disrupts iron homeostasis by competing with Fe for carrier proteins (Palit et al., 1994; Hu et al., 2021). At the cellular level, Co stress promotes the generation of reactive oxygen species (ROS), hydroxyl radicals (·OH), and hydrogen peroxide (H_2_O_2_), resulting in decreased chlorophyll (Chl) and carotenoid (Car) contents, increased malondialdehyde (MDA) and proline levels, and altered antioxidant enzyme activities (Azarakhsh et al., 2015). Furthermore, excessive Co can impair nutrient translocation, water uptake, and root development, leading to chlorosis, necrosis, reduced nodule formation in legumes, and ultimately decreased biomass and crop yield (Akeel and Jahan, 2020).

**Figure 1:**
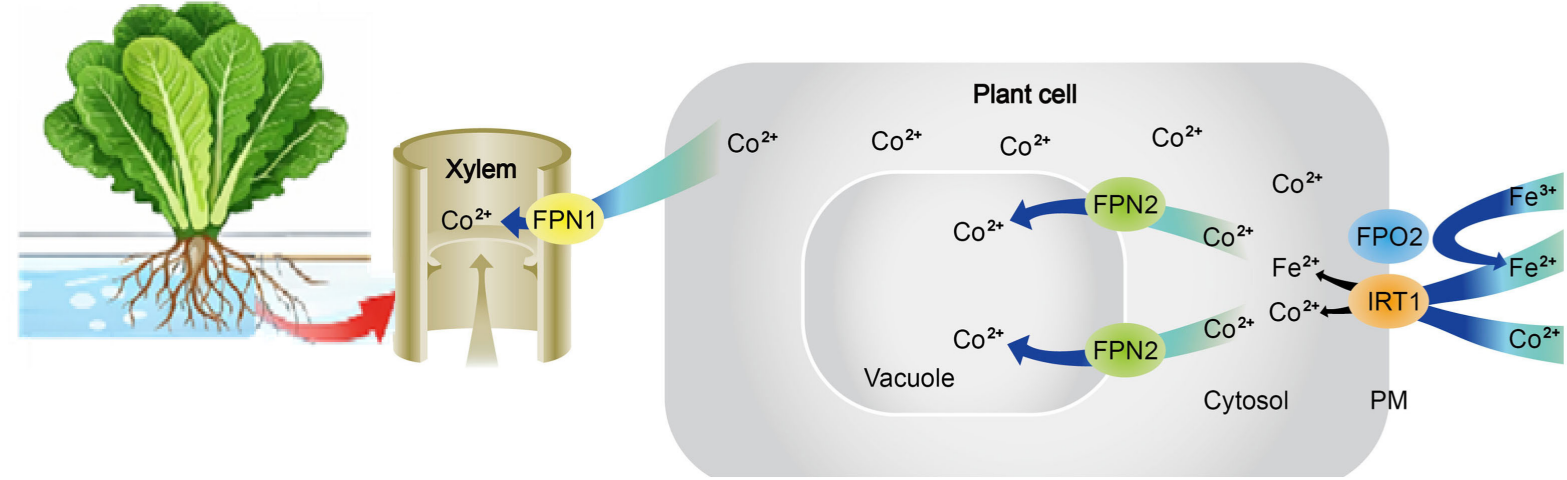
Proposed conceptual schematic model illustrating the modulatory role of nitric oxide (NO) in cobalt-induced stress responses in lettuce plants. The model is adapted from Hu et al. (2021) and modified based on the experimental findings of the present study.

Co uptake and transport are closely linked to those of other divalent cations (Fe²^+^, Ni²^+^, Zn²^+^) and involve coordinated processes in absorption, translocation, and sequestration. Co²^+^ ions are absorbed into root epidermal cells via iron transporter IRT1, followed by distribution through ferroportins FPN1 and FPN2. FPN2 sequesters Co²^+^ in vacuoles of root cells, while FPN1 mediates xylem loading. In the xylem, Co²^+^ binds with ligands such as citrate, histidine, methionine, or nicotinamide for transport to aerial organs, as summarized in the conceptual framework presented in **Figure 1**, which was adapted from previously published models of Co transport and stress responses in plants (Hu et al., 2021) and expanded to incorporate the experimental findings of the present study.

Nitric oxide (NO) is a gaseous free radical and a key signaling molecule in plants. Due to its short lifespan, small molecular size, and ability to rapidly traverse biological membranes, NO is involved in plant responses to abiotic stresses, including heavy metal toxicity. Under metal stress conditions, NO plays a central role in maintaining cellular redox homeostasis by regulating the balance between reactive oxygen species (ROS) production and antioxidant defense systems (Corpas et al., 2011; Hasanuzzaman et al., 2018). NO has been reported to enhance the activities of antioxidant enzymes such as CAT, APX, and SOD, thereby limiting oxidative damage to membranes and cellular components (Gill and Tuteja, 2010). Due to its gaseous nature, NO is commonly applied exogenously via donor compounds such as SNP or S-nitroso-N-acetylpenicillamine (SNAP), which release NO into the rooting medium (Ramamurthi and Lewis, 1997; Beligni and Lamattina, 2001). In addition, NO can mitigate metal toxicity through chelation, compartmentalization, and modulation of ion transport processes, ultimately reducing metal bioavailability within sensitive tissues (Yang et al., 2016; Kaya et al., 2019). Furthermore, NO-mediated post-translational modifications, particularly S-nitrosylation, regulate stress-responsive proteins and gene expression, contributing to enhanced stress tolerance in plants under adverse environmental conditions (Zhou et al., 2021).

Exogenous NO has been reported to enhance plant tolerance to various abiotic stresses, including heavy metal toxicity, salinity, drought, and temperature extremes, mainly by scavenging ROS and activating antioxidant defense systems (Panda et al., 2011; Mostofa et al., 2014; Bai et al., 2015; Yang et al., 2016). Therefore, the present study aimed to evaluate whether exogenous NO application via the roots could modulate antioxidant defenses in two lettuce varieties under Co stress and whether this treatment could mitigate Co-induced toxicity.

## 2 Materials and methods

### 2.1 Plant materials and experimental design

The study was conducted in a greenhouse under natural light conditions at Arslanbey Campus, Kocaeli, Turkiye (40°40′47″N, 30°01′37″E) in 2023. Three-week-old seedlings of lettuce (*Lactuca sativa* L.): curly (var. *crispa*) and Romaine (var. *longifolia*), obtained from a local supplier (PAK FIDE, Yalova, Turkiye), were used in the experiment.

Seedlings at the 3–4 leaf stage were acclimatized by irrigating with a modified Hoagland nutrient solution at varying concentrations over seven days: four days with quarter-strength and four days with half-strength solution. Seedlings that reached the 5–6 leaf stage were transferred into two-liter polyethylene containers (one plant per container) filled with perlite as an inert medium in a hydroponic system.

Plants were then subjected to four different treatments using full-strength modified Hoagland solution for 28 days: (i) Control (nutrient solution only), (ii) Co (200 µM Co as CoSO_4_·7H_2_O), (iii) SNP (200 µM sodium nitroprusside [Na_2_(Fe(CN)_5_NO)·2H_2_O]; Sigma Aldrich, St. Louis, MO, USA), (iv) Co+SNP (200 µM Co + 200 µM SNP). The SNP solutions were freshly prepared for this study and protected from light to minimize photodecomposition. SNP treatments were applied under reduced light conditions, and the nutrient solutions were renewed regularly to ensure consistent NO availability.

The experiment followed a completely randomized factorial design with three replicates per treatment. During the experimental period, average greenhouse conditions were 27 °C (day)/18 °C (night) temperature and 63% relative humidity.

Modified Hoagland solution contains 5 mM calcium nitrate tetrahydrate [Ca(NO_3_)_2_×4H_2_O], 5 mM potassium nitrate (KNO_3_), 2 mM magnesium sulfate heptahydrate (MgSO_4_×7H_2_O), 1 mM potassium di-hydrogen phosphate (KH_2_PO_4_), 45.5 µM boric acid (H_3_BO_3_), 44.7 µM iron sulfate heptahydrate (FeSO_4_×7H_2_O), 30 µM sodium chloride (NaCl), 9.1 µM manganese sulfate monohydrate (MnSO_4_×H_2_O), 0.77 µM zinc sulfate heptahydrate (ZnSO_4_×7H_2_O), 0.32 µM copper sulfate pentahydrate (CuSO_4_×5H_2_O), 0.10 µM ammonium molybdate tetrahydrate [(NH_4_)_2_Mo_7_O_24_×4H_2_O], and 54.8 µM ethylene diamine tetraacetic acid dihydrate disodium salt (Na_2_EDTA×2H_2_O) (Hoagland and Arnon, 1950). Throughout the experimental period, the pH of the solution was maintained at 6.0.

### 2.1 Sampling and harvest of plants

Plants subjected to Co and SNP treatments were harvested after 28 days of treatment under controlled greenhouse hydroponic conditions. The total plant growth period, including the initial establishment phase before treatment, was approximately 8–9 weeks. At the end of the 28-day treatment period, plants were harvested for biomass determination and subsequent analyses.

For biomass determination, the aerial parts (leafy stems) were carefully cut at the growth medium surface, and their fresh weights (FWs) were measured using a double-precision analytical balance. Root systems were gently separated from the perlite substrate, thoroughly cleaned, and weighed to determine root fresh weights.

All plant tissues were first washed with tap water to remove adhering particles and potential surface contaminants, followed by three rinses with deionized water. Samples were then dried in a forced-air oven at 70°C until a constant weight was achieved. After cooling to room temperature in a desiccator, shoot and root dry weights (DWs) were recorded.

The dried plant materials were finely ground into a homogeneous powder using a laboratory mill. The powdered samples were stored in airtight containers and subsequently used for elemental and physiological analyses to evaluate the effects of Co and SNP treatments.

### 2.2 Determination of photosynthetic pigments

Photosynthetic pigments were measured in younger, fully expanded, healthy leaves just before the harvest. For this, fresh leaf samples (0.25 g) were finely chopped and then extracted with 10 mL of 90% acetone (v/v) using a homogenizer (Heidolph DIAX 900, Kelheim, Germany). The resulting extract was filtered, and the absorbance was recorded for Chl a, Chl b, total Chl, and carotenoid (Car) content at wavelengths of 645, 663, 652, and 470 nm, respectively, using a spectrophotometer (Shimadzu UV-1201, Kyoto, Japan). The concentrations of photosynthetic pigments were calculated according to the method outlined by Lichtenthaler (1987).

### 2.3 Determination of membrane damage

Membrane permeability (MP) in fresh leaves was determined by using the electrical conductivity (EC, %) method as described by Yan et al. (1996). For measurement, fresh leaves were washed, and then a standard sample was taken out using a disc, cut into 1 cm pieces, and placed in a beaker containing 10 mL of deionized water. The leaf samples were immersed at 30 °C for 3 h, and then the EC of the solution was measured. After boiling the samples for 2 min, their conductivity was measured again when the solution was cooled to room temperature. The percentage of MP was calculated as follows:


$$\text{MP }\left(\text{EC},\%\right)=\frac{\text{C}1}{\text{C}2}\times 100$$

where: C1 and C2 are the electrolyte conductivities measured before and after boiling, respectively.

Lipid peroxidation was determined by measuring the malondialdehyde (MDA) content, a byproduct of lipid peroxidation, following the report of Hodges et al. (1999). In brief, mature leaf samples (0.25 g) were homogenized in 5 mL of 0.1% trichloroacetic acid (TCA), and the homogenate was centrifuged at 5000 g for 5 minutes. 4 mL of 20% TCA containing 0.5% thiobarbituric acid (TBA) was added to 1 mL of the supernatant. The mixture was heated in a boiling water bath at 95°C for 15 minutes and then rapidly cooled in an ice bath. After centrifugation at 10,000 g for 5 minutes, the supernatant was used for MDA analysis using a spectrophotometer. The absorbance at 532 nm was recorded and corrected for nonspecific absorbance at 600 nm. The MDA concentration was determined using an extinction coefficient of ϵ = 155 mM cm^-^¹.

### 2.4 Determination of hydrogen peroxide content and proline accumulation

The H_2_O_2_ content in the leaves was extracted and quantified following the method of Mukherjee and Choudhuri (1983). Leaf samples (0.25 g) were homogenized using a homogenizer in 5 mL of cold acetone and then filtered. A 1 mL aliquot of the extract was mixed with 4 mL of a titanium dioxide (TiO_2_) reaction solution containing TiO_2_ (0.06%, w/v), K_2_SO_4_ (0.6%, w/v), and H_2_SO_4_ (10%, v/v), followed by the addition of 5 mL concentrated ammonia (NH_3_) solution. The mixture was centrifuged at 10,000 g for 5 minutes. The yellow color intensity of the supernatant was measured at 415 nm. The H_2_O_2_ content was calculated using a standard curve prepared with H_2_O_2_ concentrations ranging from 100 to 1000 nmol.

Free proline was extracted from 0.25 g of fresh leaf tissue, homogenized with 5 mL of 3% (w/v) sulfosalicylic acid at 4°C, and quantified using the ninhydrin reagent, as described by Bates et al. (1973).

### 2.5 Enzyme extraction and assay

For the extraction and assay of enzymes, fully mature leaves (1.0 g) were mashed using a homogenizer with 5 mL of extraction buffer (100 mM Na-phosphate buffer, pH 7.5) containing 0.5 mM EDTA-Na_2_, maintained at 4°C. Then, 1 mM ascorbic acid was incorporated into the extraction buffer, as the enzyme is unstable without ascorbate to stabilize ascorbate peroxidase activity (Shigeoka et al., 2002). The resulting homogenate was then centrifuged at 10,000 g for 5 minutes. The supernatant was carefully collected for enzyme activity assays. All colorimetric measurements, including enzyme activity assessments, were carried out using a spectrophotometer at 25°C.

Catalase (CAT; EC 1.11.1.6) activity was measured by preparing a reaction mixture (2.5 mL per 0.2 mL of supernatant, pH 7) containing 50 mM KH_2_PO_4_ and 1.5 mM H_2_O_2_. The rate of H_2_O_2_ decomposition was monitored by observing the decrease in absorbance at 240 nm over 1 minute (Chance and Maehly, 1955). The activity was then calculated using the extinction coefficient (ϵ = 40 mM cm^-^¹) for H_2_O_2_.

Ascorbate peroxidase (APX; EC 1.11.1.11) activity was determined by incubating a reaction solution (3.0 mL per 0.1 mL of supernatant, pH 7) containing 50 mM KH_2_PO_4_, 0.05 mM ascorbic acid, 0.1 mM EDTA-Na_2_, and 1.5 mM H_2_O_2_. The activity was quantified by monitoring the decrease in ascorbate, reflected by the change in absorbance at 290 nm over 1 minute (Nakano and Asada, 1981). The extinction coefficient (ϵ = 2.8 mM cm^-^¹) for ascorbate was used for calculation.

### 2.6 Determination of Co concentrations and accumulations

For the determination of Co concentrations, 0.5 g of each dried shoot or root sample was ashed in a muffle furnace at 500 °C for 6 hours. After cooling, the resulting ash was dissolved in 5 mL of 0.1 M hydrochloric acid (HCl) solution, following the procedure described by Miller (1998). The Co concentrations were subsequently quantified using inductively coupled plasma–optical emission spectrometry (ICP-OES; Perkin Elmer Optima 2100 DV, Waltham, MA, USA).

The bio-concentration factor (BCF) and total accumulation rate (TAR) of Co were calculated according to the methods of Shi et al. (2010); Cikili et al. (2016), respectively, using the following equations:


$${\text{BCF}}_{\text{Co}}=\frac{{\left[\text{Co}\right]}_{\text{shoot or root}}}{{\left[\text{Total Co}\right]}_{\text{solution}}}$$

where: [Co]_shoot_ and [Co]_root_ respect the Co concentrations in shoot and root tissues, respectively, and the [total Co] concentration is added to the modified Hoagland nutrient solution.


$$\begin{array}{l} {\text{TAR}}_{\text{Co}}\left({\text{μg g}}^{-1}\;{\text{DW growth day}}^{-1}\right)\\ =\frac{\left({\left[\text{Co}\right]}_{\text{shoot}}\times {\text{DW}}_{\text{shoot}}\right)+({\left[\text{Co}\right]}_{\text{root}}\times {\text{DW}}_{\text{root}})}{\text{growth day}\times \left({\text{DW}}_{\text{shoot}}+{\text{DW}}_{\text{root}}\right)} \end{array}$$

where: [Co]_shoot_ and [Co]_root_ denote the Co concentrations in shoot and root tissues, respectively.

### 2.7 Statistical analysis

Our study was conducted in three replications according to a randomized plot design. The normality of data distribution was evaluated using the Shapiro–Wilk test, and based on the test results, statistical analysis were performed using analysis of variance (ANOVA) with MINITAB software (Version 16; Minitab Corp., State College, Pennsylvania, USA). For multiple comparisons among treatments, including control, SNP, and Co applications, mean values were separated using Duncan’s Multiple Range Test (DMRT) at the 5% significance level (α = 0.05). The levels of statistical significance are indicated as (*) *p* < 0.05, (**) *p* < 0.01, (***) *p* < 0.001, and (ns) not significant.

Pearson correlation coefficient (r) analysis was conducted separately for each lettuce variety using n=12 observations per variety (four treatments × three replications) to examine relationships among physiological, biochemical, and accumulation parameters in roots, shoots, and leaves. To minimize the risk of false-positive correlations arising from multiple testing, *p* values were adjusted using the Benjamini–Hochberg false discovery rate (FDR) correction. Correlation analyses were performed for exploratory purposes, and the observed relationships should be interpreted as indicative associations rather than evidence of causality.

The present findings highlight the complex interplay between Co toxicity, antioxidant regulation, and genotype-dependent responses in lettuce under controlled hydroponic conditions. While the experiment was designed as a factorial arrangement involving lettuce variety and treatment, statistical analyses were conducted separately for each array to emphasize within-variety treatment responses. As a result, the main effects of variety and the variety × treatment interaction were not formally tested. Therefore, comparisons between curly and Romaine lettuce should be interpreted as indicative trends rather than statistically confirmed interaction effects. Future studies employing two-way ANOVA or generalized linear models that explicitly incorporate interaction terms will be required to rigorously validate genotype-dependent differences in Co tolerance and SNP responsiveness.

## 3 Results

### 3.1 Plant growth

The effects of Co, SNP, and Co+SNP applications on the biomass production of lettuce varieties are presented in **Table 1**. Compared with the control, Co and Co+SNP treatments significantly reduced the shoot fresh weight (FW) of curly lettuce by 39.9% and 53.4%, respectively. The corresponding reductions in root FW were 37.0% and 38.9%. In contrast, the effects of the SNP alone on both shoot and root FW of curly lettuce were insignificant.

**Table 1:** The effects of Co and SNP on biomass production (FW and DW) of lettuce varieties.

Applications	Fresh weight (g pod^-1^)		Dry weight (g pod^-1^)	
	Shoot	Root	Shoot	Root
Curly lettuce				
Control	21.66 ± 0.76 a	5.24 ± 0.17 a	1.49 ± 0.05 a	0.35 ± 0.01 a
Co	13.01 ± 0.47 b	3.30 ± 0.11 b	1.22 ± 0.03 ab	0.29 ± 0.01 ab
SNP	20.27 ± 0.64 a	4.64 ± 0.20 a	1.55 ± 0.07 a	0.32 ± 0.01 a
Co+SNP	10.10 ± 0.28 b	3.30 ± 0.09 b	0.88 ± 0.02 b	0.23 ± 0.01 b
*F*-Value	12.60**	8.11**	4.60*	6.07*
Romaine lettuce				
Control	21.29 ± 0.53 b	9.20 ± 0.43 b	2.59 ± 0.11 a	0.66 ± 0.02 ab
Co	11.62 ± 0.43 c	5.55 ± 0.45 c	1.51 ± 0.07 b	0.54 ± 0.04 b
SNP	24.13 ± 0.40 a	13.30 ± 0.38 a	2.29 ± 0.02 a	0.77 ± 0.02 a
Co+SNP	15.19 ± 0.39 c	8.56 ± 0.27 b	1.61 ± 0.06 b	0.51 ± 0.02 b
*F*-Value	13.66**	9.56**	15.80**	3.65^ns^

Values are means ± SE (n = 3). Different letters within the same column for each variety indicate significant differences according to DMRT (α < 0.05). *F*-values represent the ANOVA test statistics for treatment effects. * and ** indicate significance at *p* < 0.05 and *p* < 0.01, respectively; ns, non-significant.

Similarly, Co application markedly decreased the shoot and root FW of Romaine lettuce by 45.4% and 39.7%, respectively. Co+SNP treatment resulted in a further reduction of 28.7% and 7.0% in shoot and root FW, respectively. Conversely, the SNP application alone significantly enhanced the shoot and root FW of Romaine lettuce by 13.3% and 46.6%, respectively (**Table 1**).

Regarding dry weight (DW), both shoot and root DWs of curly lettuce tended to decline under Co treatment, whereas SNP application showed no significant effect. Co+SNP treatment caused a marked decrease in shoot and root DWs by 40.9% and 34.3%, respectively. In Romaine lettuce, Co and Co+SNP applications significantly decreased the shoot DW by 41.7% and 37.8%, respectively, while the effect of SNP alone was not significant. The root DW of Romaine lettuce showed a decreasing trend under Co and Co+SNP treatments, but increased by 16.7% with SNP application (**Table 1**).

### 3.2 Photosynthetic pigment contents

**Figure 2:**
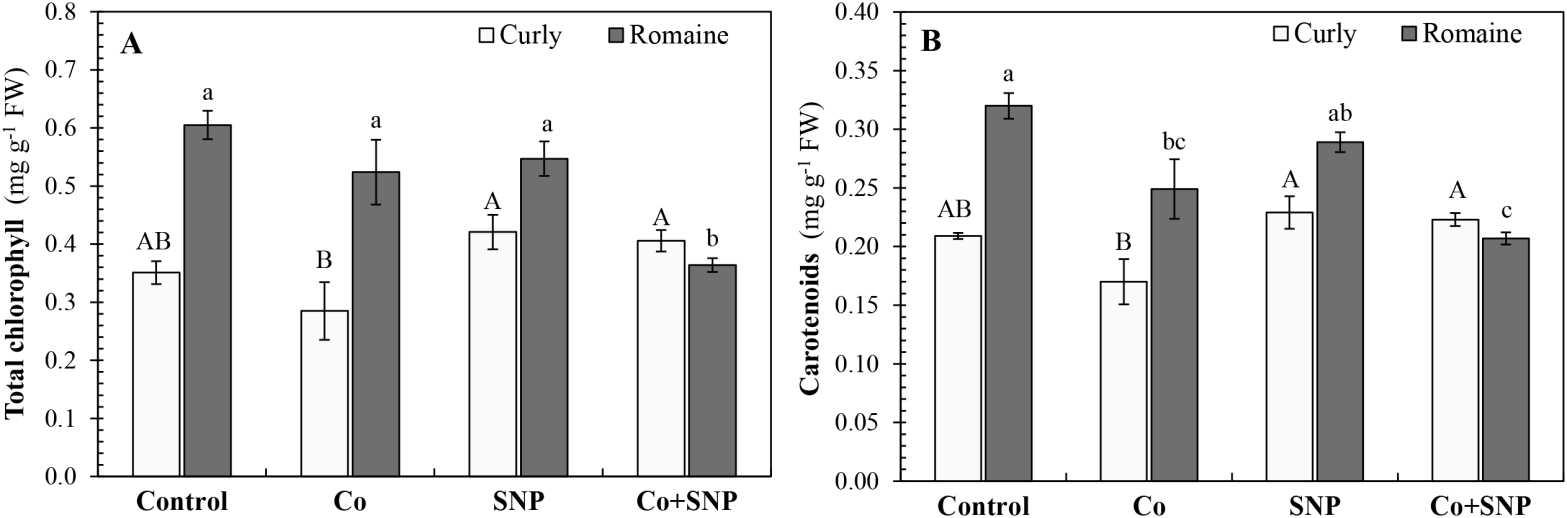
The effects of the Co and SNP on **(A)** total chlorophyll and **(B)** carotenoid contents of lettuce varieties. Values are means ± SE (n=3). Different letters indicate significant differences according to DMRT (*p* < 0.05).

The effects of the treatments on photosynthetic pigments are presented in **Figure 2**, in comparison with the control. In curly lettuce, the total chlorophyll (TChl) content showed a decreasing trend under Co treatment, whereas SNP and Co+SNP applications tended to increase TChl levels. In Romaine lettuce, the effects of Co and SNP treatments on TChl were not statistically significant; however, Co+SNP treatment caused a pronounced reduction of 39.8% compared with the control (**Figure 2A**).

In curly lettuce, the carotenoid (Car) content showed a decreasing trend under Co treatment, whereas SNP and Co+SNP applications tended to enhance this parameter. In contrast, Romaine lettuce exhibited a significant decline in Car content under Co and Co+SNP treatments, by 22.2% and, 35.3%, respectively (**Figure 2B**).

### 3.3 Membrane damage

**Figure 3:**
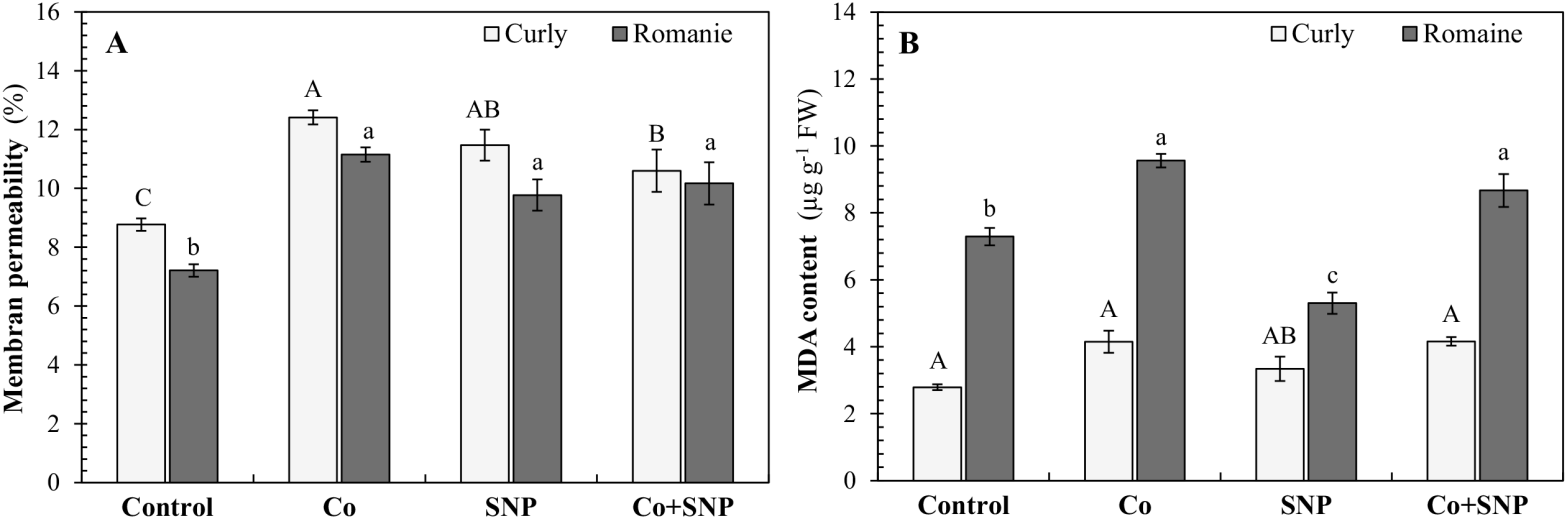
The effects of the Co and SNP on **(A)** membrane permeability and **(B)** MDA contents of lettuce varieties. Values are means ± SE (n=3). Different letters indicate significant differences according to DMRT (*p* < 0.05).

**Figure 3** illustrates the membrane permeability (MP) and lipid peroxidation (malondialdehyde, MDA) levels in the leaves of lettuce varieties. Compared with the control, all treatments (Co, SNP, and Co+SNP) markedly increased MP values in both varieties. In curly lettuce, MP increased by 41.5%, 30.8%, and 20.9%, while in Romaine lettuce, the respective increases were 54.6%, 35.5%, and 41.1% (**Figure 3A**).

Regarding lipid peroxidation, both Co and Co+SNP treatments significantly elevated MDA content in curly lettuce by 48.7% and 49.1%, respectively, compared with the control. Similarly, these treatments increased MDA levels in Romaine lettuce by 31.1% and in curly lettuce by 18.9%. Interestingly, while SNP treatment caused a slight increase in MDA content in curly lettuce, it resulted in a substantial reduction of 37.0% in Romaine lettuce (**Figure 3B**), indicating differential oxidative responses between the two varieties.

### 3.4 Hydrogen peroxide and proline accumulation

**Figure 4:**
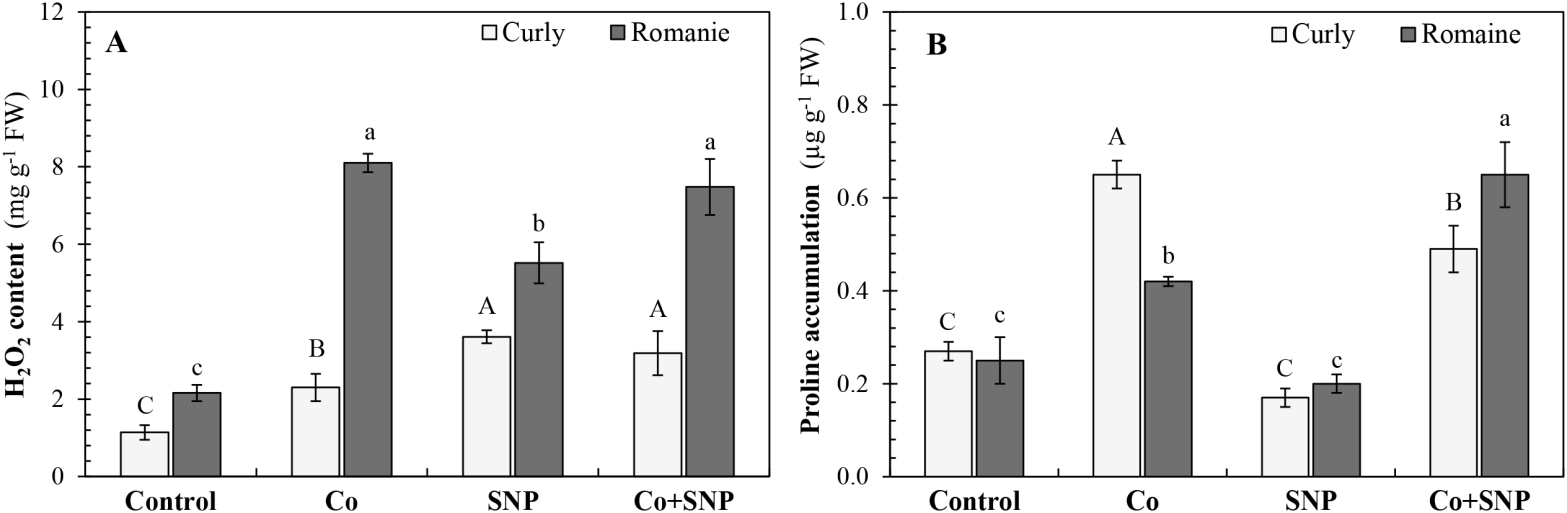
The effects of the Co and SNP on **(A)** H_2_O_2_ content and **(B)** proline accumulation of lettuce varieties. Values are means ± SE (n=3). Different letters (capital letters, curly lettuce; lowercase Romaine lettuce) indicate significant differences according to DMRT (*p* < 0.05).

As shown in **Figure 4**, all treatments caused a marked increase in hydrogen peroxide (H_2_O_2_) content in both lettuce varieties. In curly lettuce, Co, SNP, and Co+SNP treatments increased H_2_O_2_ levels by 2.0-, 3.2-, and 2.8-fold compared with the control, respectively. Similarly, these treatments elevated H_2_O_2_ content in Romaine lettuce by 3.8-, 2.6-, and 3.5-fold, respectively (**Figure 4A**).

In contrast, the effect of SNP treatment on proline accumulation was not statistically significant in either lettuce variety. However, Co and Co+SNP treatments significantly enhanced proline accumulation by 2.4- and 1.8-fold in curly lettuce and by 1.7- and 2.4-fold in Romaine lettuce, respectively (**Figure 4B**).

### 3.5 Antioxidant enzymes

**Figure 5:**
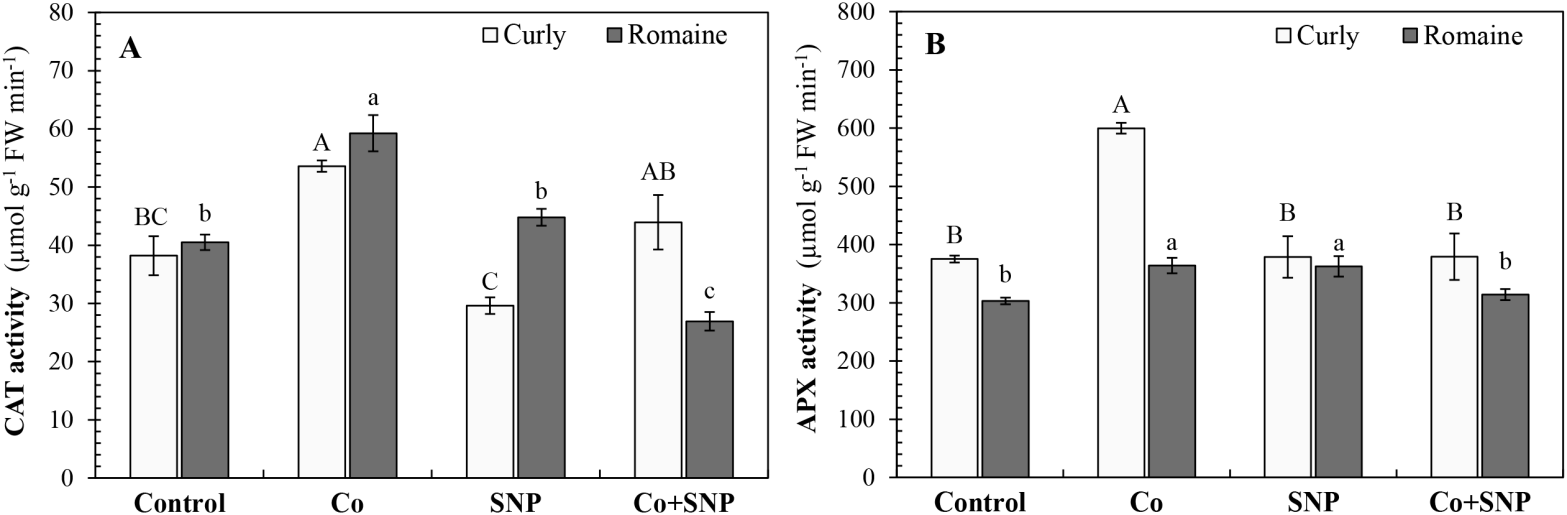
The effects of the Co and SNP on **(A)** CAT and **(B)** APX activity of lettuce varieties. Values are means ± SE (n=3). Different letters (capital letters, curly lettuce; lowercase Romaine lettuce), indicate significant differences according to DMRT (*p* < 0.05).

As shown in **Figure 5**, catalase (CAT) activity in curly lettuce was markedly enhanced by Co treatment, showing a 40.3% increase compared with the control. While SNP treatment slightly reduced CAT activity, Co+SNP treatment tended to increase it. In Romaine lettuce, Co application significantly increased CAT activity by 46.3%, whereas the Co+SNP treatment resulted in a 33.5% decrease (**Figure 5A**).

Regarding ascorbate peroxidase (APX) activity, Co treatment increased enzyme activity by 59.6% in curly lettuce and by 20.1% in Romaine lettuce. The effect of SNP treatment on APX activity was not significant in curly lettuce, but it caused a noticeable increase of 19.7% in Romaine lettuce. Co+SNP treatment had no significant effect on APX activity in either variety (**Figure 5B**).

### 3.6 Co concentrations and accumulations

**Table 2** shows that Co concentrations in both shoots and roots were significantly elevated by Co treatment compared with the control. Shoot and root Co concentrations tended to increase under SNP treatment in both lettuce varieties. Co+SNP treatment significantly enhanced shoot Co concentrations in both varieties, whereas a significant increase in root Co concentration was observed only in Romaine lettuce.

**Table 2:** Effects of cobalt (Co) and sodium nitroprusside (SNP) on Co concentrations, bio-concentration factor (BCF), and total accumulation rate (TAR) in shoots and roots of two lettuce varieties. BCF represents the ratio of Co concentration in plant tissue to that in the growth medium, indicating the efficiency of Co uptake. TAR expresses the daily rate of Co accumulation in plant tissues (µg g^−1^ DW day^−1^).

Applications	Co concentrations (µg g^-1^)		BCF of Co		TAR of Co (µg g^-1^ DW day^-1^)
	Shoot	Root	Shoot	Root	
Curly lettuce					
Control	0.18 ± 0.04 c	5.13 ± 0.30 c	17.30 ± 4.55 b	513.2 ± 29.7 b	0.20 ± 0.04 b
Co	67.32 ± 3.68 b	1447.82 ± 193 a	5.71 ± 0.32 b	122.8 ± 16.4 c	36.56 ± 9.23 a
SNP	0.49 ± 0.10 c	11.97 ± 0.60 c	49.07 ± 9.99 a	1197.1 ± 60.0 a	0.41 ± 0.06 b
Co+SNP	77.10 ± 3.84 a	683.02 ± 33.2 b	6.54 ± 0.33 b	58.0 ± 2.8 c	11.83 ± 1.34 b
*F*-Value	245.56^**^	49.16^**^	13.66^**^	230.22^**^	14.37^*^
Romaine lettuce					
Control	0.98 ± 0.03 c	4.21 ± 0.12 b	98.30 ± 3.20 b	421.50 ± 11.6 b	0.82 ± 0.03 b
Co	28.86 ± 3.65 b	904.80 ± 45.8 a	2.45 ± 0.31 c	76.80 ± 3.89 b	51.29 ± 4.65 a
SNP	1.52 ± 0.16 c	29.01 ± 2.13 b	151.73 ± 16.3 a	2900.6 ± 213.0 a	3.76 ± 0.31 b
Co+SNP	56.86 ± 1.06 a	965.14 ± 62.9 a	4.82 ± 0.09 c	81.9 ± 5.34 b	69.96 ± 4.01 a
*F*-Value	196.06^***^	186.19^***^	78.51^***^	163.02^***^	11.42^**^

Values are means ± SE (n = 3). Different letters within the same column for each variety indicate significant differences according to DMRT (α < 0.05). F-values represent the ANOVA test statistics for treatment effects. *, **, and *** indicate significance at *p* < 0.05, *p* < 0.01, and *p* < 0.001, respectively.

The BCF of Co in both shoots and roots increased significantly with SNP treatment. In curly lettuce, shoot and root BCF values increased 2.8- and 2.3-fold, respectively, while in Romaine lettuce, the increases were 1.5- and 6.9-fold. In contrast, Co and Co+SNP treatments caused a notable decrease in shoot BCF in Romaine lettuce and root BCF in curly lettuce.

The TAR of Co increased markedly under Co and Co+SNP treatments in both lettuce varieties. Interestingly, SNP treatment decreased the TAR of Co by 68.7% in curly lettuce but slightly increased it by 8.7% in Romaine lettuce, compared with the Co-treated plants.

### 3.7 Correlation analyses

**Figure 6:**
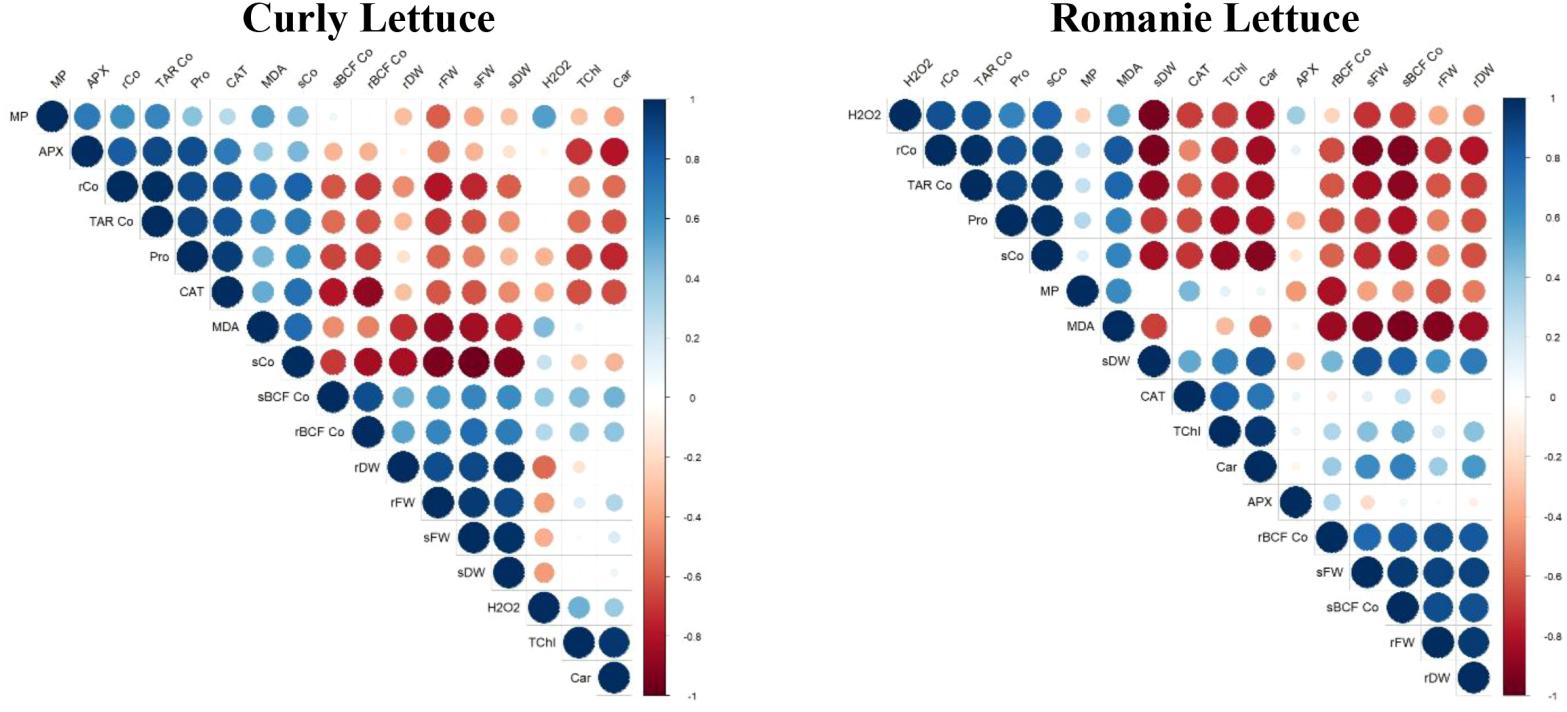
The correlation matrix illustrates the relationships among parameters measured in the roots and shoots of lettuce varieties. In the matrix, the circles’ color and size reflect the strength and direction of the correlations: blue circles indicate positive correlations, while red circles denote negative correlations. Larger circles correspond to stronger correlation coefficients (*MP*, membrane permeability; *APX*, ascorbate peroxidase; *CAT*, catalase; *r*, root; *s*, shoot; *Co*, cobalt; *TAR*, total accumulation rate; *Pro*, proline; *MDA*, malondialdehyde; *BCF*, bio-concentration factor; *DW*, dry weight; *FW*, fresh weight; *H_2_O*_2_, hydrogen peroxide; T*Chl*, total chlorophyll; *Car*, carotenoids).

Pearson correlation analysis revealed significant relationships among growth, pigment contents, stress markers, and Co accumulation parameters (**Figure 6A**). Biomass production parameters (shoot and root FW and DW) were strongly and positively correlated with each other in both lettuce varieties (r > 0.853). TChl and Car contents also exhibited a significant positive correlation in curly (r = 0.952) and Romaine lettuce (r = 0.953).

In contrast, growth parameters were negatively correlated with oxidative stress markers (MDA, H_2_O_2_), proline content, and Co accumulation (shoot and root Co concentrations). Strong positive correlations were observed among oxidative stress indicators (MDA, H_2_O_2_), antioxidant enzyme activities (CAT, APX), and Co accumulation parameters (sCo, rCo, sBCF, rBCF) (r > 0.753). Notably, Co concentration in the root (rCo) concentration displayed a strong linear relationship with TAR, sCo, proline, CAT, and MDA, with correlation coefficients ranging from 0.737 to 0.986 (**Figures 6A, B**).

## 4 Discussion

Lettuce is a widely consumed leafy vegetable, rich in polyphenolic compounds such as phenolic acids and flavonoids, which confer health benefits and play a key role in enhancing plant tolerance to environmental stresses (Ayuso-Calles et al., 2020). In recent years, exogenous NO has been reported to mitigate heavy metal toxicity in plants by enhancing antioxidant defense systems, reducing oxidative damage, and promoting growth under stress conditions (Xiong et al., 2009; Wang et al., 2010; Panda et al., 2011).

### 4.1 Effects of Co and SNP on growth and pigments

Based on our experimental results, a conceptual schematic model summarizing the modulatory role of nitric oxide in cobalt-induced stress responses and Co accumulation in lettuce plants is presented in **Figure 1**.

Excess Co exposure markedly impaired lettuce growth, as reflected by significant reductions in shoot and root fresh and dry weights in both varieties (**Table 1**). These growth limitations are closely associated with Co-induced oxidative stress and disruption of membrane integrity (**Figure 3A**), which compromise photosynthetic efficiency (**Figure 2**) and metabolic balance. Consistent with this interpretation, reductions in total chlorophyll and carotenoid contents were observed under Co stress, likely resulting from the inhibition of key enzymes involved in chlorophyll biosynthesis and pigment stability (Sree et al., 2015). Similar Co-induced growth and pigment reductions have been reported in several crop species, including barley, tomato, and mung bean (Jayakumar et al., 2007; Gopal et al., 2003; Chatterjee and Chatterjee, 2005; Agnihotri et al., 2014).

The application of SNP differentially modulates growth parameters between lettuce varieties. While SNP alone significantly enhanced growth in Romaine lettuce, no significant improvement was observed in curly lettuce (**Table 1**). Under Co+SNP treatment, pigment contents were markedly restored in curly lettuce, compared to Co alone. In contrast, Romaine lettuce exhibited a more pronounced improvement in growth parameters, suggesting genotype-physiological stress responses.

The differential response of curly and Romaine lettuce to SNP application indicates a genotype-specific sensitivity to NO-related regulation. In the present study, SNP increased shoot FW and reduced lipid peroxidation (MDA) in Romaine lettuce, whereas similar improvements were not observed in curly lettuce, compared to the control (**Table 1**; **Figure 3B**). Such contrasting responses may be attributed to differences in NO sensitivity, uptake efficiency, or downstream signaling capacity between genotypes, as NO is known to function as a signaling molecule whose effectiveness varies depending on genetic background (Corpas and Barroso, 2015). Romaine lettuce may possess a more responsive NO signaling network that allows more effective modulation of antioxidant defenses and maintenance of membrane integrity under Co stress (**Figure 3A**). In contrast, the higher Co accumulation observed in curly lettuce may have imposed a greater oxidative burden, potentially exceeding the protective threshold of SNP-derived NO and limiting its beneficial effects (Xiong et al., 2010; Hasanuzzaman et al., 2018). These findings highlight the importance of genotype-dependent metal handling and redox regulation in determining the effectiveness of NO-based mitigation strategies.

Although SNP is widely used as an NO donor in plant studies, it is well-documented that SNP decomposition may also generate cyanide- and iron-cyanide-related species, which can independently affect oxidative processes (Mur et al., 2013). In the absence of additional controls such as alternative NO donors or NO scavengers, it is not possible to attribute the observed physiological responses exclusively to NO (Arasimowicz-Jelonek and Floryszak-Wieczorek, 2011). Therefore, the effects observed in the present study are more accurately described as SNP-derived NO-associated responses rather than strictly NO-associated mechanisms. The significant increase in MP percentage with SNP application can be evaluated within this framework (**Figure 3A**). Nevertheless, the consistency of these responses with previous SNP-based studies supports a central role of NO signaling in modulating antioxidant activity and stress tolerance under Co exposure.

### 4.2 Cobalt accumulation and translocation

Accumulation of Co in shoots and roots increased significantly under Co treatment (**Table 2**), consistent with prior reports showing that Co supplementation enhances metal uptake and translocation (Jayakumar et al., 2007; Agnihotri et al., 2014). The SNP application alone had no significant effect on biomass in curly lettuce, but slightly enhanced growth in Romaine lettuce. Co+SNP treatment improved total chlorophyll and carotenoid contents in curly lettuce, compared to Co alone, whereas in Romaine lettuce, these effects were less pronounced (**Figure 2**). The increase in carotenoids may protect the photosynthetic apparatus from ROS, while NO may stabilize pigments and maintain photosynthetic efficiency by alleviating oxidative membrane damage (Panda et al., 2011; Stra et al., 2023).

Co accumulated predominantly in roots rather than shoots, indicating a protective sequestration strategy that limits metal toxicity in aerial tissues. Root confinement of heavy metals has been widely reported as a tolerance mechanism that restricts translocation and protects photosynthetically active tissues (Collins et al., 2010; Hu et al., 2021; Klink et al., 2013). SNP application altered Co distribution patterns in a variety-dependent manner. Notably, Co+SNP reduced root Co accumulation in curly lettuce, compared to Co alone, whereas this effect was less evident in Romaine lettuce, reflecting differences in metal transport regulation and NO responsiveness. The NO may enhance shoot development and antioxidant defense, modulate cell wall relaxation, influence membrane fluidity, and improve nutrient transport, collectively affecting Co distribution (Kaya et al., 2019; Ozfidan-Konakci et al., 2020). Pearson correlation analysis supported these findings, showing strong positive correlations between shoot FW and chlorophyll contents (r = 0.980 with TChl, r = 0.969 with Car) under Co+SNP treatment (**Figure 6B**).

Variations in BCF and TAR further support the role of NO in modulating Co uptake and distribution. NO has been shown to influence ion transport, membrane fluidity, and cell wall dynamics, thereby affecting heavy metal accumulation efficiency and translocation (Xiong et al., 2009; Corpas et al., 2011; Cikili et al., 2016; Sharma and Agrawal, 2006).

### 4.3 Oxidative stress and antioxidant responses

Co toxicity primarily impaired lettuce growth by inducing oxidative stress and subsequently disrupting cellular membrane integrity. Elevated levels of MDA and H_2_O_2_, along with increased membrane permeability and proline accumulation (**Figures 3**, **4**), suggest that the Co-induced redox imbalance triggered lipid peroxidation and compromised membrane stability (Apel and Hirt, 2004). These interconnected physiological disturbances collectively disrupted nutrient transport, cellular signaling, and metabolic processes, ultimately resulting in growth inhibition in both lettuce varieties. Proline accumulation acts as an osmoprotectant and metal chelator, contributing to stress tolerance and maintaining turgor pressure (Kaur and Asthir, 2015; Wang et al., 2015).

**Figure 5** showed that antioxidant enzyme activities (CAT and APX) increased under Co stress, reflecting an adaptive response aimed at detoxifying excessive ROS. The CAT enzyme plays a key role in scavenging H_2_O_2_, while APX participates in ascorbate-dependent ROS detoxification (Gill and Tuteja, 2010). SNP-mediated NO supply modulated these antioxidant responses, enhancing enzyme activities in some tissues while showing variable effects across genotypes, likely due to differences in NO sensitivity, endogenous redox buffering capacity, and Co accumulation levels (Groß et al., 2013; Laspina et al., 2005).

Compared with Co-applied plants, the decrease in Co concentration observed particularly in roots after Co+SNP application, and the modulation of CAT and APX activities, can be explained by known NO-related signaling mechanisms (**Table 2**, **Figure 5**). Xiong et al. (2010) reported that NO regulates the activity of membrane transporters and ion channels through redox-based modifications, thereby limiting excessive metal influx into plant cells. In parallel, NO fine-tunes antioxidant enzyme activities through post-translational regulation, including S-nitrosylation, thereby enabling efficient ROS scavenging without excessive enzyme activation (Corpas and Barroso, 2015). Such balanced regulation of antioxidant defenses is essential for maintaining redox homeostasis under metal stress and has been widely reported as a key function of NO signaling in plants (Hasanuzzaman et al., 2018).

In Romaine lettuce, SNP application effectively reduced MDA content compared to the control, coinciding with improved shoot growth (**Figure 3B**). In contrast, the higher Co accumulation observed in curly lettuce may have exceeded the protective threshold of NO, thereby limiting its beneficial effects despite enhanced antioxidant enzyme activity (**Table 2**). Such genotype-dependent NO responses have been reported previously and highlight the complex interaction between metal load, redox regulation, and NO signaling efficiency (Groß et al., 2013; Manai et al., 2014).

Beyond the descriptive changes observed in antioxidant enzyme activities, SNP-derived NO plays a regulatory role at multiple physiological levels. NO functions as a signaling molecule that modulates antioxidant enzyme activities, contributing to effective ROS scavenging and redox homeostasis under Co stress (Corpas and Barroso, 2015). In addition, NO influences ion transport and metal distribution by regulating membrane properties and transporter activity, thereby limiting excessive Co influx or promoting its compartmentalization. NO also contributes to pigment stability by protecting chloroplast membranes from oxidative damage, supporting the maintenance of photosynthetic pigments under stress conditions.

NO also plays a critical role in regulating ion transport and metal distribution by affecting membrane fluidity, transporter activity, and cell wall properties, which can restrict excessive Co influx or facilitate its compartmentalization in less sensitive tissues (Xiong et al., 2010). Such regulation contributes to improved ionic balance and reduced metal-induced toxicity.

Furthermore, NO contributes to the stabilization of photosynthetic pigments by protecting chloroplast membranes and preserving the structural integrity of the photosynthetic apparatus under oxidative stress conditions. By limiting ROS-mediated degradation of chlorophylls and carotenoids, NO supports pigment preservation and photosynthetic efficiency during heavy metal exposure (Hasanuzzaman et al., 2018). Together, these coordinated regulatory roles provide a mechanistic explanation for the NO-associated enhancement of antioxidant activity, modulation of ion movement, and pigment stability observed in the present study.

### 4.4 Integrated stress response

Pearson correlation analysis revealed strong negative correlations between growth parameters and Co accumulation or oxidative stress markers, whereas oxidative stress indicators showed positive correlations with antioxidant enzyme activities (**Figures 6A, B**). Root Co concentration showed strong linear relationships with TAR, shoot Co concentration, proline content, CAT activity, and MDA levels, highlighting its close association with physiological stress responses.

In-depth, Co-induced toxicity impaired growth, pigment stability, and membrane integrity through oxidative stress–mediated mechanisms. SNP-associated NO application partially alleviated these effects by reinforcing antioxidant defenses, stabilizing membranes, and modulating Co distribution in a genotype-dependent manner. The contrasting responses of curly and Romaine lettuce highlight the importance of genetic variation in determining NO responsiveness and metal tolerance, underscoring the potential of NO-based strategies for improving crop resilience under heavy metal stress.

On the other hand, it should be emphasized that the concentrations of Co and SNP used in this study represent a high-dose hydroponic stress scenario rather than environmentally realistic field conditions. Although Co toxicity in plants has been reported to occur at much lower concentrations, the use of 200 µM Co in the present experiment was intentionally selected to induce a clear and reproducible stress response, allowing the assessment of genotype-dependent tolerance and the modulatory effects of SNP under severe metal stress. Therefore, the findings should be interpreted within the context of acute hydroponic exposure and should not be directly extrapolated to agricultural field conditions. Future studies incorporating dose–response designs and field-relevant Co levels will be necessary to evaluate the applicability of these responses under realistic cultivation scenarios.

It is important to consider the implications of Co accumulation in edible tissues from a food safety perspective. Lettuce is consumed primarily for its leaves, and the elevated Co concentrations observed in shoot tissues under Co and Co+SNP treatments raise potential concerns regarding consumer exposure. Leafy vegetables are known to efficiently accumulate heavy metals, which may increase the risk of metal entry into the food chain (Peralta-Videa et al., 2009). Although Romaine lettuce exhibited lower shoot Co accumulation compared with curly lettuce, this does not imply suitability for food production in Co-contaminated environments. Rather, the observed varietal differences reflect relative physiological tolerance and metal handling capacity under controlled hydroponic conditions. Cultivation of edible leafy vegetables in metal-contaminated systems may pose health risks even when plant growth appears unaffected, emphasizing the need to distinguish between plant stress tolerance and food safety (Rascio and Navari-Izzo, 2011). Consequently, the present findings should not be interpreted as a recommendation for growing lettuce for consumption in Co-contaminated environments, but rather as a basis for understanding genotype-dependent metal responses and for informing risk assessment, controlled cultivation, or non-food applications such as phytoremediation under strictly regulated conditions.

Overall, Co-induced growth inhibition in lettuce is primarily associated with oxidative stress–mediated membrane damage rather than isolated physiological effects. The concurrent increases in reactive oxygen species, lipid peroxidation, and membrane permeability observed under Co stress highlight a tightly interconnected response cascade leading to impaired growth. Exogenous NO supplied via SNP modulated this cascade by reinforcing antioxidant defenses and maintaining redox balance, although its effectiveness varied between genotypes. Romaine lettuce exhibited greater responsiveness to SNP application, whereas higher Co accumulation in curly lettuce appeared to limit the protective capacity of NO. These findings emphasize the importance of genotype-dependent redox regulation and metal handling in determining plant tolerance to Co stress.

## 5 Conclusion

Co exposure induced pronounced oxidative stress, membrane damage, and growth inhibition in lettuce, accompanied by elevated Co accumulation in both roots and shoots. Romaine lettuce generally exhibited lower shoot Co accumulation and a more stable physiological response than curly lettuce, reflecting genotype-dependent differences in metal handling under controlled hydroponic conditions.

When applied together with Co, SNP partially alleviated cobalt-induced stress relative to Co treatment alone, as evidenced by moderated MP, reduced lipid peroxidation (MDA), and improved redox regulation, particularly in Romaine lettuce. In contrast, the limited response of curly lettuce may be associated with its higher Co load, which likely exceeded the protective capacity of SNP-derived NO.

Importantly, the accumulation of Co in edible shoot tissues raises concerns regarding food safety. Therefore, the present findings should not be interpreted as a recommendation for cultivating lettuce for consumption in Co-contaminated environments. Rather, this study provides mechanistic insight into genotype-dependent stress responses under high-dose hydroponic Co exposure and may inform risk assessment, controlled cultivation strategies, or non-food applications such as phytoremediation under strictly regulated conditions.

## Data Availability

The original contributions presented in the study are included in the article/**Supplementary Material**. Further inquiries can be directed to the corresponding author.
