# Supplementary material for: Modulatory role of nitric oxide in cobalt-induced stress in two lettuce (lactuca sativa l.) varieties: a physiological approach
Source: Front Plant Sci. 2026 Feb 20;17:1731303. doi: 10.3389/fpls.2026.1731303 (PMC12963352; doi:10.3389/fpls.2026.1731303)
Supplement: Supplementary file 1 [file DataSheet1.pdf]

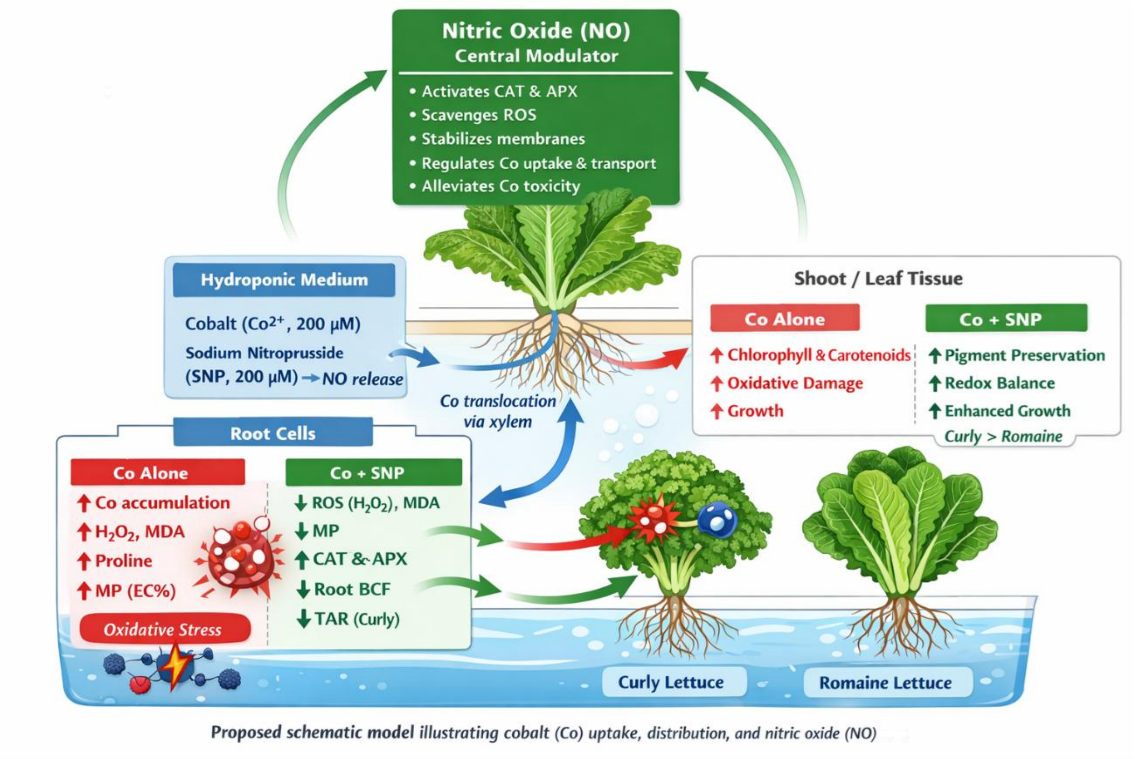

Graphical Abstract. Schematic illustration of cobalt (Co) uptake, translocation, and toxicity in lettuce plants and the modulatory role of nitric oxide (NO) supplied via sodium nitroprusside (SNP). Excess Co induces oxidative stress, membrane damage, and growth inhibition through enhanced reactive oxygen species (ROS) production and lipid peroxidation. Exogenous NO activates antioxidant enzymes (CAT and APX), stabilizes cellular membranes, regulates Co uptake and distribution, and alleviates Co-induced toxicity in a genotype-dependent manner, with differential responses observed between curly and Romaine lettuce.
